# Research and Development of Medical Countermeasures for Emerging Infectious Diseases, China, 1990–2022

**DOI:** 10.3201/eid3101.230638

**Published:** 2025-01

**Authors:** Jiyan Ma, Yang Yang, Yangmu Huang

**Affiliations:** Author affiliation: Peking University School of Public Health, Beijing, China

**Keywords:** medical countermeasures, vaccines, infectious diseases, communicable diseases, emerging diseases, research and development, China, mpox, COVID-19, SARS-CoV-1, SARS-CoV-2

## Abstract

Since the severe acute respiratory syndrome outbreak in 2003, China has invested substantial efforts in promoting scientific and technological advances for medical countermeasures against high-threat pathogens. The examination of China’s landscape identifies progress and gaps in research and development (R&D) and also highlights management and regulatory issues that should be of concern to other countries. Our study examined the current state of R&D of medical countermeasures in China during 1990–2022. The findings showed a robust and diversified pipeline responding quickly to disease outbreaks and policy changes. However, proactive and highly innovative candidates are limited, and a large proportion of vaccines and drugs stagnate at the early development stage. A paradigm shift involving a preemptive R&D agenda and persistent investment, innovative technology development, and accelerated research translation is urgently needed to prepare for the next pandemic.

The unprecedented outbreaks of mpox and COVID-19 reminded the world that the global research and development (R&D) system was unprepared to tackle emerging infectious diseases (EIDs) ahead of outbreaks. A collective approach for developing effective medical countermeasures, including vaccines, therapeutic drugs, and diagnostic devices, is urgently needed to avert large-scale crises and strengthen global health security (*1*). As a high-risk country for indigenous and imported pathogens, China has put substantial effort into promoting scientific and technological advances for medical countermeasures with regulatory reforms and strategies (*2*). Improvements in China’s ability to detect, prevent, and control EIDs are of global interest. However, because of lacking or insufficient data sources, information on the progress of R&D in China is limited (*3*,*4*). To evaluate to what extent the current pipeline addresses EIDs with pandemic and epidemic potential, our study comprehensively analyzed publicly available information on R&D efforts in China, including the development pipeline, clinical trials, and R&D activities during 1990–2022.

## Methods

### Disease Scope

The scope of EIDs included in this analysis is mainly based on the R&D Blueprint of the World Health Organization (WHO) (*5*). As a global strategy and plan of action, the Blueprint prioritized a list of pathogens that will most likely cause the next epidemic and require proactive R&D activities (*5*). We included all pathogens identified or discussed in the Blueprint and others recognized in peer-reviewed literature as posing public health risks to China since 1990, except those with a wide range of R&D pipelines, funding flows, or global elimination or eradication programs (i.e., HIV/AIDS, malaria, tuberculosis, viral hepatitis, COVID-19, smallpox, and Guinea worm disease). A total of 63 diseases were identified and grouped into 3 categories: viral, bacterial, and parasitic and other groups (Appendix).

### Search Strategy and Eligibility

We identified all originally China-developed vaccine, therapeutic, and diagnostic candidates for human use and phase I–III clinical trials during January 1, 1990–September 20, 2022, on the PharmCube and Yaozhi databases. Those 2 databases cover the most comprehensive information of its kind on the entire lifecycle of pharmaceutical products developed in China from multiple sources, such as the National Medical Products Administration, Center for Drug Evaluation, and Chinese Clinical Trial Registry, and have been widely used by industry, academia, and government for pharmaceutical analysis (*6*–*9*). We retrieved the profiles of candidates and clinical trials by defining pathogen or disease name, related MeSH terms, or cataloged synonyms. We classified candidates into the following types: pathogen/disease, development phase, product type, technology platform, developer type, R&D type, and regulatory pathway. We classified clinical trials by pathogen/disease, study phase, study type, location, sponsor and collaborator type, and first posted year. Two reviewers (J.M. and Y.Y.) manually verified data with parameters of candidate and pathogen names across the Center for Drug Evaluation Annual Drug Review Report, Chinadrugtrials.org.cn, ClinicalTrials.gov, Pharmsnap database, company and WHO websites, media and press releases, and other sources. A third researcher (Y.M.) reviewed discordant information until a consensus was reached.

To capture the portfolio and public funding of basic research, we also searched for research projects funded by the National Natural Science Foundation of China (NSFC) on its Big Data Knowledge Management Service Portal during January 1, 1990–December 31, 2019 (when the portal was updated). The funding was converted into US dollars using the exchange rate for the year of award from the International Monetary Fund. By searching government websites and PKUlaw (China’s legal and normative database), we systematically collected national-level policy documents related to EID R&D, covering laws, regulations, procedures, plans, strategies, norms, and guidelines.

Eligible candidates included vaccines, innovative therapeutics, and diagnostic devices initially developed for EIDs by developers in China. We excluded those developed by foreign enterprises but licensed to or manufactured in China, biosimilar drugs, improved new drugs, and generic drugs. We deemed clinical trials eligible if they were registered for diagnosis, treatment, prevention, or control of EIDs by developers in China. We excluded trials applied by foreign developers but carried out in China. We included NSFC projects if they specifically mentioned in research objectives or expected outcomes the advancement of knowledge and new technologies for detecting, preventing, controlling, or treating EIDs. We excluded projects related to EIDs but outside the scope of medical countermeasures (i.e., projects focused on etiology, physiology, ecology, or public health). We identified policies if they explicitly mentioned in the theme or content accelerating or promoting R&D for diagnosing, treating, preventing, or controlling EIDs or in the context of public health emergencies.

## Results

### Overview of EID Pipeline in China

As of September 20, 2022, at least 118 vaccines, 52 therapeutics, and 285 diagnostic devices were in China’s pipeline, covering a total of 36 EIDs (Appendix). Diseases with the most medical countermeasure candidates were dengue fever (49 candidates), enterovirus A71 infection (43 candidates), and rabies (35 candidates). In contrast, Crimean-Congo hemorrhagic fever, Rift Valley fever, Marburg virus disease, and African trypanosomiasis were the least represented diseases; only 1 candidate each was under early development.

Collectively, 41.5% of vaccines and 36.5% of therapeutics were in the preclinical phase, substantially more than were in phase I–III clinical trials (18.6% of vaccines and 23.1% of therapeutics). Of the 317 medical countermeasures approved for market, 249 (78.5%) were diagnostic devices, largely held by dengue fever–focused devices approved for export (44 of 45); approved vaccines (14.8%, 47) and therapeutics (6.6%, 21) were represented by Japanese encephalitis (11 vaccines), shigellosis, necrotizing cellulitis, and necrotizing fasciitis (5 drugs each). The WHO also prequalified 2 of the approved vaccines: the live-attenuated Japanese encephalitis vaccine (SA 14-14-2) in 2013 and the inactivated influenza (H1N1) vaccine (split virion) in 2015 (*10*).

### R&D Characteristics of EID Candidates

**Figure 1:**
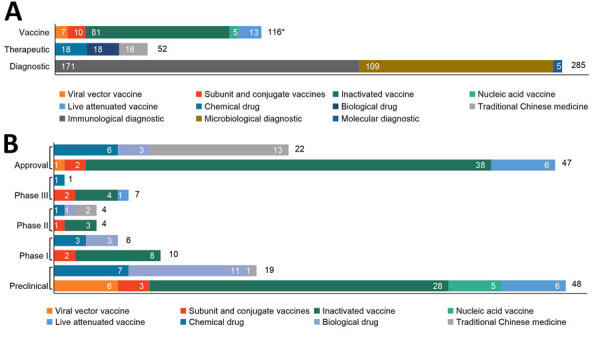
Pipeline of medical countermeasures for emerging infectious diseases by technology platform, China, 1990–2022. A) Distribution of medical countermeasures by technology platform; B) distribution of vaccines and therapeutics by technology platform and development phase. Because of limited information, 2 vaccine candidates in preclinical and phase II stages could not be classified by technology platform.

For the technology platform, inactivated vaccines (69.8%) and immunological diagnostics (60.0%) accounted for the largest percentages of each portfolio (Figure 1, panel A). Chemical and biologic drugs contributed an equal share of therapeutic portfolio, 34.6%. However, approved drugs largely consisted of traditional Chinese medicines (13 of 21) (Figure 1, panel B). The most advanced therapeutic candidate was a bispecific antibody for rabies (GR1801) in phase III clinical trial. It has demonstrated broad-spectrum neutralizing activity against naturally occurring rabies virus glycoprotein and pseudo-typed rabies virus, and trials were expected to be completed by July 2024 (ClinicalTrials.gov no. NCT05846568) (*11*). Several candidates based on emerging technologies (e.g., mRNA vaccine, monoclonal antibody) were at preclinical and phase I stages.

**Figure 2:**
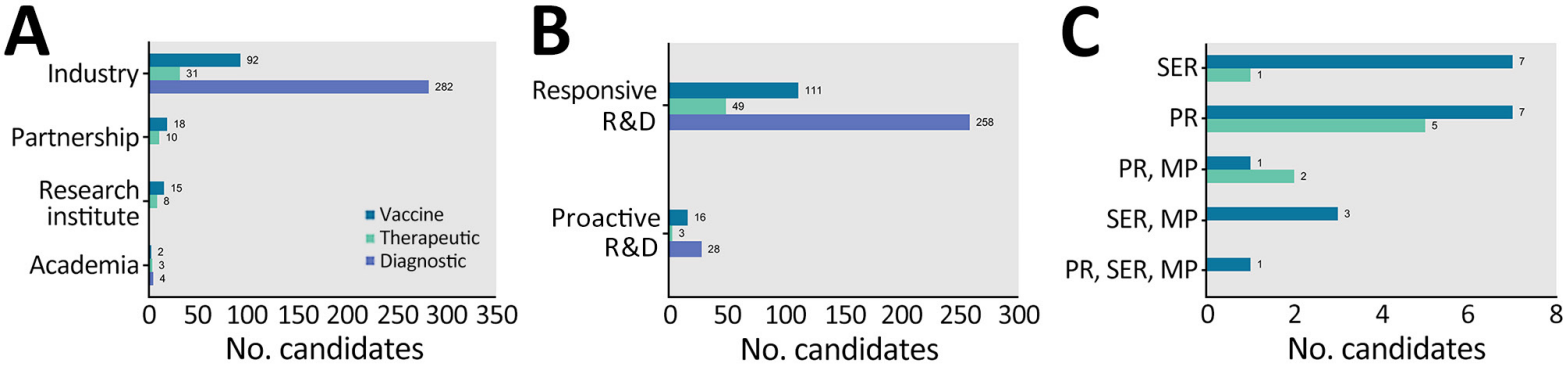
Pipeline of medical countermeasures for emerging infectious diseases by developer type, R&D type, and regulatory pathway, China, 1990–2022. Partnership indicates that the candidate was jointly developed by >2 developers. Responsive and proactive R&D were differentiated on the basis of the time between the occurrence of disease in China and the earliest R&D of candidate. A) Distribution of medical countermeasures by developer type and countermeasure type. B) Distribution of medical countermeasures by R&D type. C) Distribution of medical countermeasures by regulatory pathway. MP, Major Program of National Science and Technology; PR, Priority Review; R&D, research and development; SER, Special Examination Review.

Assessment of candidate developers showed the key involvement of private industry in developing 78.0% of vaccines (92 candidates), 59.6% of therapeutics (31 candidates), and 98.9% of diagnostics (282 candidates) (Figure 2, panel A). The vaccine and therapeutic pipelines were concentrated in 2 pharmaceutical companies (Sino Biopharmaceutical [https://www.sinobiopharm.com] and Jiangsu Kanion Pharmaceutical [https://kanion.en.made-in-china.com]), accounting for 27.9% of vaccines and 13.2% of therapeutics. In contrast, the diagnostic pipeline is widely distributed; 81 companies were working on >1 diagnostic product for EIDs. Comparing the timeline between the occurrence of disease in China and the earliest R&D activity, ≈90% of candidates of each medical countermeasure type were responsively developed after the onset of diseases (Figure 2, panel B). A few candidates were proactively initiated in case of the appearance and transmission of influenza (H1N1), yellow fever, Ebola, Marburg virus, and West Nile virus. For regulatory pathway, the Ebola recombinant adenovirus vector-based vaccine (Ad5-EBOV) was the only product that received priority review, special examination review, and major program support simultaneously (Figure 2, panel C). It became the first domestically developed product approved for strategic national stockpile under emergency use authorization (*12*). Other candidates granted the largest number of expedited regulatory designations were vaccines for influenza (H7N9) (5 candidates) and enterovirus A71 infection (3 candidates).

### Trends of R&D Activities for EIDs

**Figure 3:**
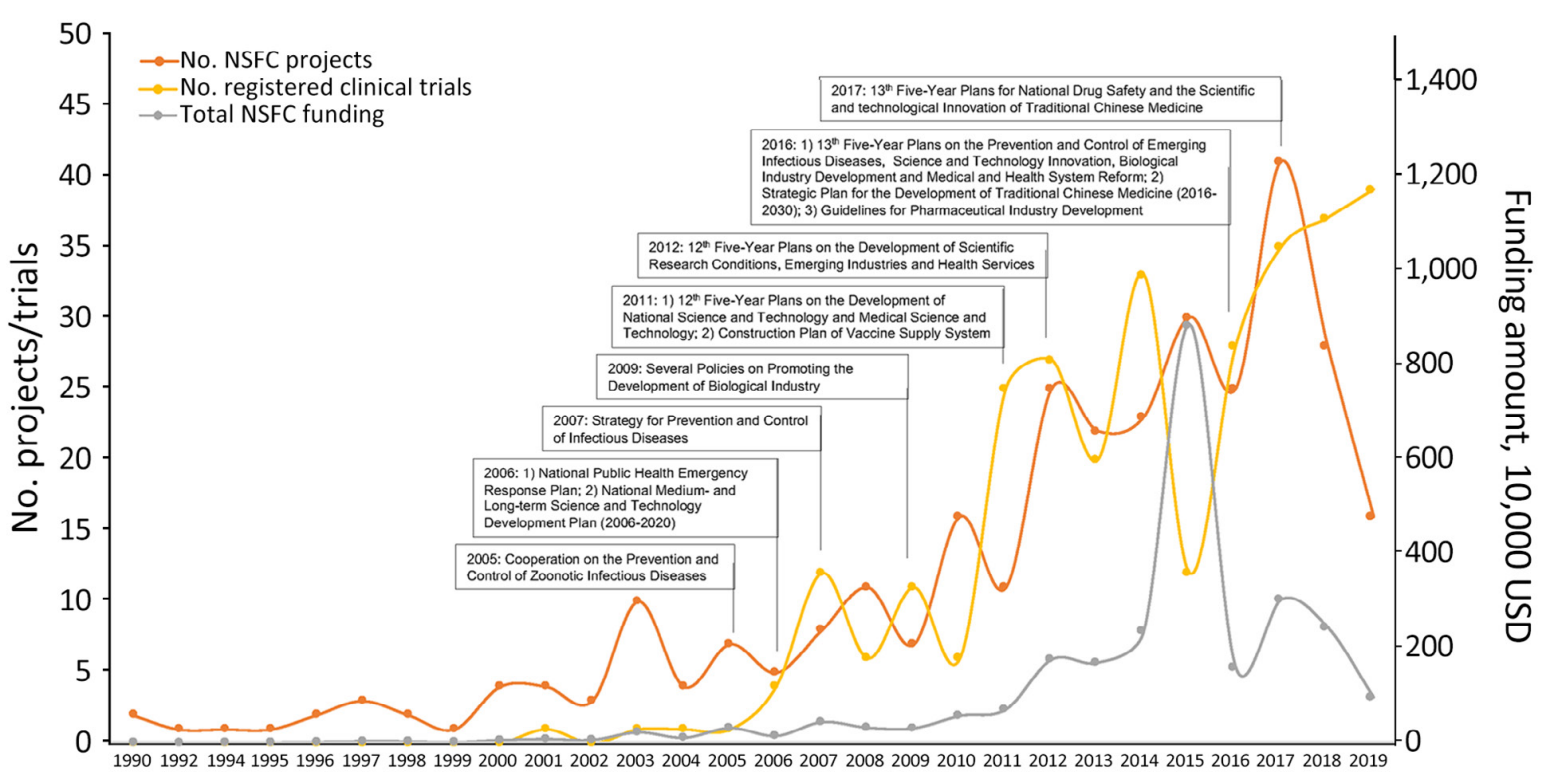
Titles and corresponding years of policy documents issued by government of China regarding research and development activities on emerging infectious diseases, 1990–2022.

During 1990–2019, China invested a total of US $26.3 million in 313 NSFC research projects to promote knowledge and develop technologies and products for detecting, preventing, and treating EIDs (Figure 3). The largest batch (33.5%, US $8.8 million) was in 2015, which included the funding of medical countermeasure development against Ebola virus disease (27.0%, US $7.1 million). The number of trials entering all stages of clinical development increased after 2005; the average annual growth rate was 59.8%. The sharp decline in 2015 could be explained by the self-inspection and verification process conducted by the National Medical Products Administration the same year, in which a large number of clinical trial applications were withdrawn or rejected to ensure data authenticity and integrity in regulatory filings (*13*). The trends of NSFC funding and projects were consistent with the timing of a series of strategic plans and policies on promoting new technology and product development for major EIDs. After 2017, momentum gradually decreased until it reached 2011 levels in 2019, indicating a swift deviation of public interest in EID research in the postoutbreak period.

**Figure 4:**
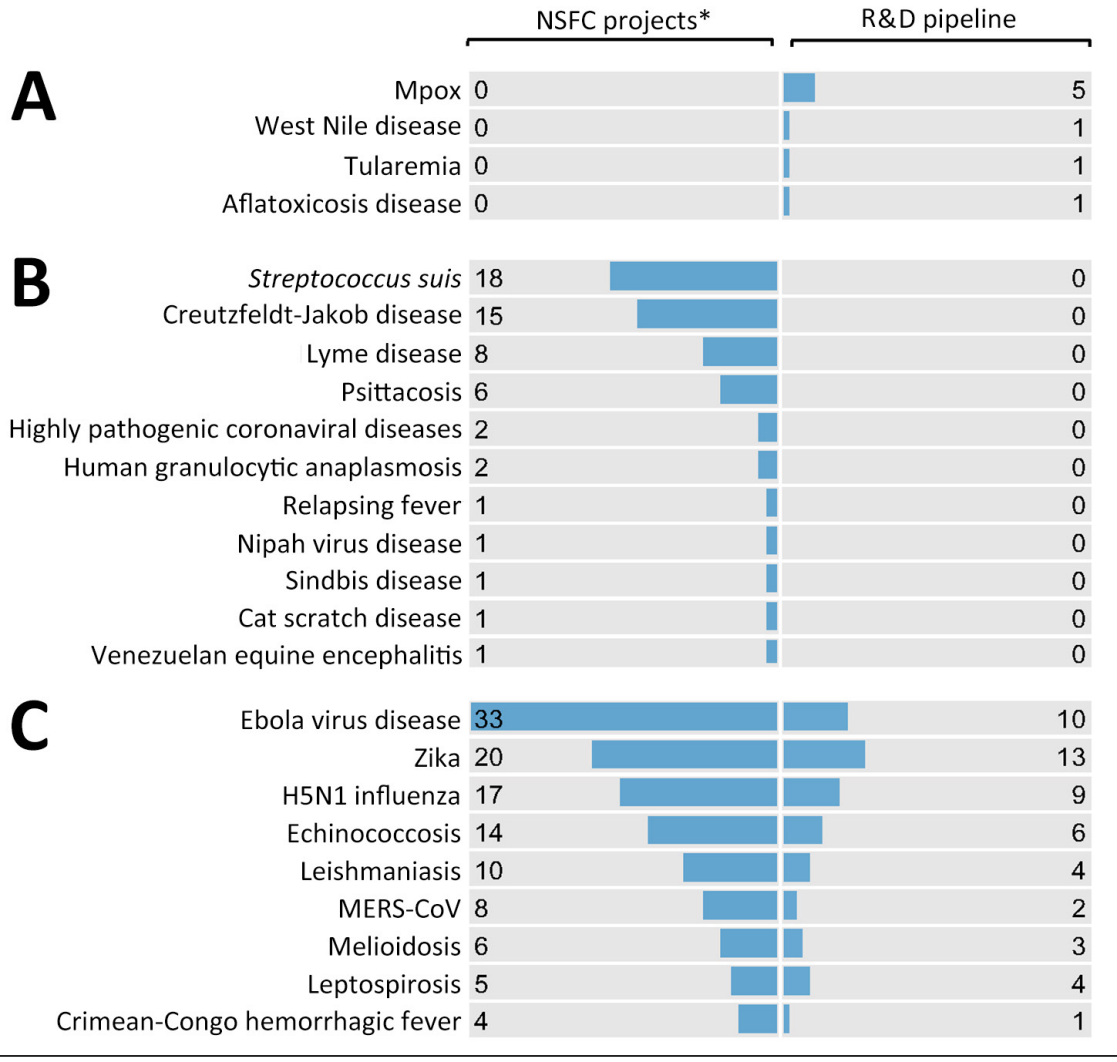
Distribution of NSFC projects and R&D pipeline by disease in study of R&D of medical countermeasures for emerging infectious diseases, China, 1990–2022. The figure shows a rough comparison between basic and applied research because NSFC projects were collected during 1990–2019 and the R&D pipeline was updated in September 2022. A) Diseases with crucial gaps in basic research indicated by lack of NSFC projects. B) Diseases with crucial gaps in applied research indicated by lack of R&D pipeline. C) Disease that might enhance translational research from basic to application include those with a large discrepancy between the number of NSFC projects and R&D pipeline (in descending order). MERS-CoV, Middle East respiratory syndrome coronavirus; NSFC, National Natural Science Foundation of China; R&D, research and development.

To understand the relative intensity of basic and applied research, we roughly compared the number of NSFC projects and R&D pipeline by each EID. The imbalanced distribution highlighted 3 groups of EIDs in need of urgent action: diseases with crucial gaps in basic research (e.g., mpox, West Nile virus, and tularemia) (Figure 4, panel A); diseases with crucial gaps in applied research (e.g., *Streptococcus suis*, Creutzfeldt-Jakob disease, and Lyme disease) (Figure 4, panel B); and diseases that might enhance translational research from basic to application (e.g., Ebola virus disease, Zika virus, and influenza [H5N1]) (Figure 4, panel C). In addition, of the 63 diseases included in the analysis, 16 have neither basic research nor R&D pipelines according to public data; included in those 16 diseases were Lassa fever and henipavirus disease, both of which were prioritized by the WHO R&D Blueprint (*5*).

## Discussion

Our study provides current and comprehensive country-level evidence on medical countermeasure development for EIDs in China. Since the lessons learned from severe acute respiratory syndrome, China has attached great importance to public health preparedness and response capacity-building. The successful development of Ebola vaccine indicates a robust national commitment and governance of R&D forces in response to global public health emergency. The pipeline, research, and clinical activities responded quickly to public policies and disease outbreaks, indicating fundamental R&D capacity and infrastructure to meet environmental changes. However, despite substantial efforts achieved over 3 decades, a major and persistent gap remains in basic research, product development, and clinical activities for most EIDs. Cutting-edge candidates are few, and most active candidates stagnated at early development phase. To better prepare for future outbreaks, urgent actions are needed to increase early R&D planning and investment in EIDs, enhance innovative technology platform development, and promote translation research from basic discovery into clinical advance.

Because the emergence of EIDs is challenging to predict and usually confined to limited geographic settings, the global R&D pipeline has lacked appropriate investment until a new pathogen shows explosive growth (*14*). Pharmaceutical companies have little market incentive to invest resources in developing medical countermeasures that are unlikely to yield comparable investment return versus other opportunities in a short time (*15*). That persistent underinvestment leads to the absence of available products to meet population need at the time of outbreak, which in turn causes increased health burden, economic losses, and social instability (*16*). Compared with oncology, the R&D hotspot in China, the pipeline for medical countermeasures for EIDs has a huge gap in terms of scale and vitality; the total number of oncology therapeutics (359) in 2020 was 6.9 times higher than the total number of EID therapeutics (52), and the proportion of candidates in phase I–III trials (71.9%) was >3.1 times that of candidates for EIDs (23.1%) (*17*). For diseases posing a major global and national threat (e.g., mpox, Lassa fever, and *Streptococcus suis*), the current pipeline is missing *>*1 of the basic, translational, and applied research stages. Most NSFC projects and candidate development were launched responsively after outbreak of disease, resulting in a funding cycle too short to produce noteworthy basic discoveries and technology breakthroughs. Our findings are consistent with studies worldwide that reported patterns of insufficient and unsustainable funding, responsive R&D activities, and weak correlation between disease burden and research investment (*3*,*18*). To overcome market failures, governments should take responsibility for developing national plans and strategies to accelerate R&D and stockpiling of medical countermeasures with persistent and proactive inputs before the next pandemic (*15*). Many developed countries, such as the United States, United Kingdom, and Australia, have already established R&D preparedness and response plans for tackling high-risk and unknown pathogens (*19*–*21*). Establishing a country-level R&D agenda could provide a cohesive framework for defining critical gaps, focusing on leading technology development, and allocating funding and resources to the highest-priority pathogens on the basis of health and R&D needs. Through the development of medical countermeasures, countries can also take advantage of their own scientific, technological, and innovative strengths for expanding into global markets. This opportunity has been evidenced in China’s mass development of diagnostic devices for dengue fever, which are partly associated with the mature technology and production capacity advantage of domestic industry, as well as by substantial demand from neighboring countries for cross-border screening and virological surveillance (*22*,*23*).

Although the current portfolio covers a range of technical routes, candidates from emerging biotechnologies are few, and licensed products mainly depend on conventional inactivated vaccines and traditional Chinese medicines. Those techniques were supported by well-understood safety profiles, mature technology, and ease of production (*24*,*25*). However, given the unpredictable, evolving, and highly contagious natures of EIDs, platform-based technology and prototype-based research approaches must be built for a more rapid and effective response to future outbreaks (*26*). For example, the platform-based technology of the mRNA and viral vector vaccine is designed to assemble genetic material coding into the platform backbone (e.g., a synthetic RNA or viral vector) for inducing immunity once the virus’s genetic information is identified (*27*,*28*). This process can achieve last-mile innovation of medical countermeasures against multiple pathogens and enable large-scale production with minimal changes to development, manufacturing, and quality control processes (*15*,*29*). For instance, the mRNA-1273 vaccine for COVID-19 (Moderna, https://www.modernatx.com) took only 66 days from gene sequencing to initiation of the first phase I clinical trial (*30*). It achieved the fastest speed in vaccine development history with proven effectiveness at preventing illness (*31*). In addition, prototype-based research can also accelerate product development by filling research gaps of viruses of related families prospectively (*32*). For instance, by leveraging knowledge gained from dengue, West Nile virus, and other flaviviruses, researchers rapidly developed animal models, immunogenicity assays, and vaccine designs against Zika virus when the outbreak started (*26*).

Our findings also highlight areas that might enhance translation from basic science to clinical application. Some EIDs (e.g., Ebola and Zika virus) are intrinsically difficult to translate because of the inability to recruit sufficient numbers of infected persons as participants. Historically, efficacy trials for severe acute respiratory syndrome and Zika virus vaccines were not completed before the pandemic/epidemic ended (*33*,*34*). In this situation, early phase I and II candidates and overseas clinical trial partners should be established in advance to quickly promote the start of phase III clinical trials in the most affected areas during emergencies (*32*). Another reason for the fragmentation between basic and applied research could be the lack of communication and collaboration among the R&D community (*35*). Although the current pipeline involves a mix of industry–research institute, industry–academia, industry–industry, and research institute–research institute partnerships, >89.0% of medical countermeasures were developed by individual companies. The high costs of late-stage product development, particularly those related to long-term clinical trials, manufacturing, and commercialization, are likely to result in market failure of financial returns from EID medical countermeasures (*36*). To cross the ever-widening gap in translational research, functional interaction and coordination mechanisms, including innovation-focused grants, should exist among funding agencies, academia, research institutes, and industry (*37*).

Our analysis is mainly limited by scarce publicly available data sources. Although great efforts were made to ensure this analysis was as complete as possible and comparable to global-level assessments, data availability and quality varied substantially across databases, diseases, medical countermeasure types, and research activities. Because of data availability, national investment in major national research programs (such as the Major National Scientific Research Programs and the National Key Scientific Research Plans) and direct institutional funding from academia and nonprofit institutions were not included in the public funding analysis, which might underestimate China’s overall public R&D efforts on EIDs. Many companies could discontinue candidate development without publicly announcing the decision and being captured by the database. This landscape assessment should be regarded as a snapshot at the time of its creation. As more information and data become available, the scale and vitality might change.

## Conclusions

Our study shows a vibrant and diversified R&D pipeline in China, which has the fundamental capacity and infrastructure to respond rapidly to public policies and disease outbreaks. However, medical countermeasures for most EIDs are insufficient or lacking. Conventional technical routes remain the main focus of development; little emphasis is placed on highly innovative technology and proactive candidates. The pipeline faces the risk of drying up, especially when a large proportion of vaccines and drugs cumulate in the early development phase. Our findings serve as a resource for ongoing portfolio management and draw attention to areas in immediate need of increased support. We hope to highlight the significance of establishing a preemptive R&D agenda and investment, promoting innovative technology development, and enhancing translation research that could be conducive to other countries.

Appendix Additional information about research and development of medical countermeasures for emerging infectious diseases, China, 1990–2022
