# Supplementary material for: Research and Development of Medical Countermeasures for Emerging Infectious Diseases, China, 1990–2022
Source: Emerg Infect Dis. 2025 Jan;31(1):e230638. doi: 10.3201/eid3101.230638 (PMC11682787; doi:10.3201/eid3101.230638)
Supplement: Appendix — Additional information about research and development of medical countermeasures for emerging infectious diseases, China, 1990–2022 [file 23-0638-Techapp-s1.pdf]

Article DOI: https://doi.org/10.3201/eid3101.230638

EID cannot ensure accessibility for supplementary materials supplied by authors. Readers who have difficulty accessing supplementary content should contact the authors for assistance.

Research and Development of Medical Countermeasures for Emerging Infectious Diseases, China, 1990–2022

Appendix

Appendix Table. List of emerging infectious diseases included in analysis

No.	Disease	Source
A) Viral		
1.1	Crimean-Congo hemorrhagic fever	2018 WHO Blueprint Shortlist (*1*)
1.2	Rift Valley Fever	2018 WHO Blueprint Shortlist (*1*)
1.3	Severe fever with thrombocytopenia syndrome	2018 WHO Blueprint Longlist (*1*)
1.4	Hantavirus disease	2018 WHO Blueprint Longlist (*1*)
1.5	Ebola virus disease	2018 WHO Blueprint Shortlist (*1*)
1.6	Marburg virus disease	2018 WHO Blueprint Shortlist (*1*)
1.7	Lassa hemorrhagic fever	2018 WHO Blueprint Shortlist (*1*)
1.8	South American hemorrhagic fever	2017 WHO Blueprint Shortlist (*2*)
1.9	Arenaviral hemorrhagic fevers	2018 WHO Blueprint Longlist (*1*)
1.10	Middle East respiratory syndrome	2018 WHO Blueprint Shortlist (*1*)
1.11	Severe acute respiratory syndrome	2018 WHO Blueprint Shortlist (*1*)
1.12	Highly pathogenic coronavirus disease	2018 WHO Blueprint Longlist (*1*)
1.13	Nipah virus disease	2018 WHO Blueprint Shortlist (*1*)
1.14	Henipaviral disease	2018 WHO Blueprint Shortlist (*1*)
1.15	Zika virus disease	2018 WHO Blueprint Shortlist (*1*)
1.16	Usutu disease	2017 WHO Blueprint Shortlist (*2*)
1.17	West Nile fever	2017 WHO Blueprint Shortlist (*2*)
1.18	Dengue	Discussed in 2018 WHO Blueprint Longlist (*1*)
1.19	Yellow fever	Discussed in 2018 WHO Blueprint Longlist (*1*)
1.20	Kyasanur forest disease	2017 WHO Blueprint Shortlist (*2*)
1.21	Enteroviruses D68 infection	2018 WHO Blueprint Longlist (*1*)
1.22	Enteroviruses A71 infection	2018 WHO Blueprint Longlist (*1*)
1.23	Chikungunya virus disease	2018 WHO Blueprint Shortlist (*1*)
1.24	Sindbis disease	2017 WHO Blueprint Shortlist (*2*)
1.25	Mayaro disease	2017 WHO Blueprint Shortlist (*2*)
1.26	Alphaviruses disease	2017 WHO Blueprint Shortlist (*2*)
1.27	Chandipura virus	2017 WHO Blueprint Shortlist (*2*)
1.28	Endemic kaposi syndrome	2017 WHO Blueprint Shortlist (*2*)
1.29	Orpouche disease	2017 WHO Blueprint Shortlist (*2*)
1.30	Monkeypox	Discussed in 2018 WHO Blueprint Longlist (*1*)
1.31	H1N1 influenza	Discussed in 2018 WHO Blueprint Longlist (*1*)
1.32	H5N1 influenza	Discussed in 2018 WHO Blueprint Longlist (*1*)
1.33	H7N9 influenza	Discussed in 2018 WHO Blueprint Longlist (*1*)
1.34	Rabies	Liu Q, Cao L, Zhu XQ (*3*)
1.35	Japanese encephalitis	Chala B, Hamde F (*4*)
1.36	Venezuelan equine encephalitis	Li X, Huang J (*5*)
B) Bacterial		
2.1	Anthrax	2017 WHO Blueprint Longlist (2)

No.	Disease	Source
2.2	Brucellosis	2017 WHO Blueprint Longlist (*2*)
2.3	Plague	2017 WHO Blueprint Longlist (*2*)
2.4	Necrotising Cellutitis & Fasciitis	2017 WHO Blueprint Long List (*2*)
2.5	Cholera	2017 WHO Blueprint Long List (*2*)
2.6	Leptospirosis	2018 WHO Blueprint Long List (*1*)
2.7	Candida Auris disease	2017 WHO Blueprint Long List (*2*)
2.8	Lyme disease	Chala B, Hamde F (*4*)
2.9	Streptococcus suis	Wang L, Wang Y, Jin S, Wu Z, Chin DP, Koplan JK, et al (*6*)
2.10	Tularemia	Xiao Q, Lv Z, Qin J (*7*)
2.11	Legionellosis	Xiao Q, Lv Z, Qin J (*7*)
2.12	Melioidosis	Xiao Q, Lv Z, Qin J (*7*)
2.13	*Escherichia coli* O157:H7	Zhang S, Luo P, Gao L (*8*)
2.14	Shigellosis	Zhu Q (*9*)
2.15	Cat scratch disease	Chen H, Ma H, Liu M, Jiang H (*10*)
2.16	Relapsing fever	Xiao Q, Lv Z, Qin J (*7*)
	C) Parasitic and other	
3.1	Leishmaniasis	2017 WHO Blueprint Longlist (*2*)
3.2	Aflatoxicosis disease	2017 WHO Blueprint Long List (*2*)
3.3	African trypanosomiasis	Li X, Huang J (*11*)
3.4	Onchocerciasis	Xiao Q, Lv Z, Qin J (*7*)
3.5	Echinococcosis	Wang X, Rainey JJ, Goryoka GW, Liang Z, Wu S, Wen L, et al (*12*)
3.6	Histoplasmosis	Xiao Q, Lv Z, Qin J (*7*)
3.7	Creutzfeldt-Jakob disease	Xiao Q, Lv Z, Qin J (*7*)
3.8	Rocky mountain spotted fever	Xiao Q, Lv Z, Qin J (*7*)
3.9	Human granulogtic anaplasmosis	Zhang S, Luo P, Gao L (*8*)
3.10	Scrub typhus	Xiao Q, Lv Z, Qin J (*7*)
3.11	Psittacosis	Xiao Q, Lv Z, Qin J (*7*)

Diseases*	Vaccine			Therapeutic			Diagnostic	
	Preclinical	Clinical	Approval	Preclinical	Clinical	Approval	Validation	Approval
Dengue	0	0	0	2	0	0	2	45
EV-A71	10	3	3	4	4	2	0	17
Rabies	6	6	8	1	2	2	2	8
Hantavirus infection	0	0	7	0	1	0	4	17
H1N1 influenza	2	0	10	0	0	1	1	15
Japanese encephalitis	7	0	11	0	0	0	3	5
H7N9 influenza	5	4	0	0	0	1	0	10
SARS	0	1	0	3	0	0	0	15
Zika	8	0	0	2	0	0	0	3
Ebola virus disease	2	0	1	1	1	0	0	5
H5N1 influenza	0	3	1	3	0	0	1	1
SFTS	1	0	0	0	0	0	1	6
Monkeypox	3	0	0	0	1	0	0	1
Chikungunya virus infection	0	0	0	0	0	0	0	4
MERS-CoV	0	0	0	1	0	0	0	1
Yellow fever	1	0	0	0	0	0	0	1
CCHF	0	0	0	0	0	0	1	0
RVF	1	0	0	0	0	0	0	0
Marburg virus disease	0	0	0	0	0	0	1	0
WNF	0	0	0	0	0	0	0	1
Shigellosis	1	1	1	1	0	5	2	21
Cholera	2	1	1	0	0	4	3	19
Brucellosis	0	0	1	0	0	0	2	13
Plague	0	1	1	0	0	0	4	8
Legionellosis	0	0	0	0	0	0	7	6
Anthrax	0	0	0	0	1	0	0	8
Necrotizing Cellulitis & Fasciitis	0	2	1	0	1	4	0	0
Leptospirosis	0	0	1	0	0	0	1	2
E. coli O157:H7	0	0	0	0	0	0	1	2
Melioidosis	0	0	0	0	0	0	0	3
Tularemia	0	0	0	0	0	0	0	1
Echinococcosis	0	0	0	0	0	0	0	6
Leishmaniasis	0	0	0	0	0	0	0	4
Onchocerciasis	0	0	0	0	1	1	0	0
African trypanosomiasis	0	0	0	1	0	0	0	0
Aflatoxicosis disease	0	0	0	0	0	0	0	1
Total	49	22	47	19	12	21	36	249

Appendix Figure. Overview of EID pipeline in China. Diseases were grouped by virus, bacteria, and parasites and others. CCHF, Crimean-Congo hemorrhagic fever; *E. coli* O157:H7, *Escherichia coli* O157:H7; EV-A71, Enterovirus A71; MERS-CoV, Middle East respiratory syndrome coronavirus; RVF, Rift valley fever; SARS, severe acute respiratory syndrome; SFTS, severe fever with thrombocytopenia syndrome; WNF, West Nile fever.

References

1. World Health Organization. The 2018 annual review of diseases prioritized under the Research and Development Blueprint informal consultation [cited 2023 Oct 20]. https://www.who.int/news-room/events/detail/2018/02/06/default-calendar/2018-annual-review-of-diseases-prioritized-under-the-research-anddevelopment-blueprint

2. World Health Organization. The 2017 annual review of diseases prioritized under the Research and Development Blueprint informal consultation [cited 2023 Oct 20]. https://cdn.who.int/media/docs/default-source/blue-print/2017-annual-review-of-diseases-prioritized-under-the-research-and-development.pdf?sfvrsn=a6654583_2

3. Liu Q, Cao L, Zhu XQ. Major emerging and re-emerging zoonoses in China: a matter of global health and socioeconomic development for 1.3 billion. Int J Infect Dis. 2014;25:65–72. PubMed https://doi.org/10.1016/j.ijid.2014.04.003

4. Chala B, Hamde F. Emerging and re-emerging vector-borne infectious diseases and the challenges for control: a review. Front Public Health. 2021;9:715759. PubMed https://doi.org/10.3389/fpubh.2021.715759

5. Li X, Huang J. High-risk emerging infectious diseases in China [in Chinese]. Chin J Zoonoses. 2018;2:182–7.

6. Wang L, Wang Y, Jin S, Wu Z, Chin DP, Koplan JP, et al. Emergence and control of infectious diseases in China. Lancet. 2008;372:1598–605. PubMed https://doi.org/10.1016/S0140-6736(08)61365-3

7. Xiao Q, Lv Z, Qin J. Analysis of the risks and hazards of the introduction of the world's important infectious diseases in China [in Chinese]. Port Health Control. 2002;7:19–24.

8. Zhang S, Luo P, Gao L. Epidemiology and strategies of key emerging infectious diseases in China [in Chinese]. Chin J of Dis Control Prev. 2012;16:892–6.

9. Zhu Q. Research status of emerging infectious diseases in pediatrics [in Chinese]. Chin J of Clin Lab Manag. 2019;7:5–10.

10. Chen H, Ma H, Liu M, Jiang H. Emerging and re-emerging vector-borne diseases: present status and strategies. Int J of Med Parasiti Dis. 2011;1:39–44.

11. Li X, Huang J. Several emerging infectious diseases existing risks to China at present [in Chinese]. Chin J Zoonoses. 2018;34:182–7.

12. Wang X, Rainey JJ, Goryoka GW, Liang Z, Wu S, Wen L, et al. Using a One Health approach to prioritize zoonotic diseases in China, 2019. PLoS One. 2021;16:e0259706. PubMed https://doi.org/10.1371/journal.pone.0259706
